# Childhood social characteristics and drug-related mortality by age 41: a register-based study of Finnish birth cohorts 1982–2004

**DOI:** 10.1093/eurpub/ckag083

**Published:** 2026-06-16

**Authors:** Sini P Laakso, Lauren Bishop, Juha Luukkonen, Lasse Tarkiainen, Pekka Martikainen

**Affiliations:** Helsinki Institute for Demography and Population Health, University of Helsinki, Helsinki, Finland; Max Planck–University of Helsinki Center for Social Inequalities in Population Health, Helsinki, Finland; Helsinki Institute for Demography and Population Health, University of Helsinki, Helsinki, Finland; Max Planck–University of Helsinki Center for Social Inequalities in Population Health, Helsinki, Finland; Helsinki Institute for Demography and Population Health, University of Helsinki, Helsinki, Finland; Max Planck–University of Helsinki Center for Social Inequalities in Population Health, Helsinki, Finland; Helsinki Institute for Demography and Population Health, University of Helsinki, Helsinki, Finland; Max Planck–University of Helsinki Center for Social Inequalities in Population Health, Helsinki, Finland; Helsinki Institute for Demography and Population Health, University of Helsinki, Helsinki, Finland; Max Planck–University of Helsinki Center for Social Inequalities in Population Health, Helsinki, Finland; Max Planck Institute for Demographic Research, Rostock, Germany

## Abstract

Attained socioeconomic position is associated with drug-related deaths, but whether social characteristics in childhood predict drug-related mortality remains unclear. We estimated differences in drug-related mortality between ages 16 and 41 by parental education, household income, and household type. We used administrative data on all Finnish residents born 1982–2004 (*N* = 1 446 548). Underlying and contributory causes of drug-related mortality (1997–2023) were drawn from Causes of Death Register records and parental education, household income and household type (age 15) were derived from administrative records. Differences in drug-related mortality by childhood social characteristics and their co-occurrence were estimated using sex-stratified Cox regression. Birth cohort differences were estimated using Kaplan-Meier failure curves. The drug-related mortality rate per 100 000 person-years for men (21.9, 95% CI: 21.1–22.8) was over 3× higher than that of women (5.7, 5.3–6.2). Among both men and women, respectively, lower parental education (basic: HR 1.86, 1.63–2.11; HR 1.78, 1.37–2.32) and non-nuclear household type (single-parent households: HR 2.10, 1.89–2.33; HR 3.06, 2.46–3.79) independently predicted higher mortality hazards net of all predictors, whereas the estimates for household income greatly attenuated. Interaction analyses between parental education and household type suggested larger relative increases in mortality hazards by lower parental education among individuals from non-nuclear families. Mortality differences were consistently observed across birth cohorts. Childhood household type and parental education independently predicted subsequent drug-related mortality among men and women. Children of lower educated parents from non-nuclear families had the highest excess risk.

## Introduction

Premature drug-induced mortality is a growing public health concern in many high-income countries [1–4]. Drug-induced mortality has slowly increased in some European countries over the past 20 years [1, 4–7], with rates above the European average reported in the Nordic countries [4, 7], and has increased exponentially in the United States [8]. Drug overdose fatalities are largely attributed to opioid and polydrug use [4, 8–10] and the increasing variety and quantity of seized substances in the European drug markets pose a pressing public health concern [2]. Although drug-induced deaths remain relatively rare at the population level in Finland, they are a substantial contributor to mortality among the young: one in four deaths among 15- to 24-year-olds in 2023 was drug-induced [7].

Previous research has established a link between social disadvantage and mortality due to drug use [11–13], with both drug-induced and drug-related mortality used as outcomes. Drug-induced mortality is often defined as poisoning (or overdose) [11, 12] or deaths where the underlying cause was a drug use disorder [13]. Drug-related mortality expands this definition to include deaths in which drug use was a contributory cause [13]. Studies typically focus on attained socioeconomic position, considering factors including educational level, earnings, labour market attachment, and residential stability. These associations are likely reciprocal, where social disadvantage may be causally linked to an increased risk of drug use, and, conversely, drug use may affect an individual’s socioeconomic prospects. Measuring social characteristics in early life may partially overcome such endogeneity problems, as parental social factors can be seen as independent of the individual’s later drug use. Furthermore, by measuring early life social characteristics, associations between these factors and risk of drug-related mortality can be estimated for younger individuals, whose own social positions have not yet been fully established.

Life course epidemiology proposes that early life circumstances significantly impact health outcomes later in life. Exposure to adverse social and physical factors during critical or sensitive periods may increase the risk of long-term health harms. Individuals from lower socioeconomic positions may also experience more stressors but have lower social support or less effective coping styles to manage them. Conversely, higher levels of socioeconomic and familial resources might be protective from harmful exposures [14]. Although survey-based studies have generally found weak or mixed associations between childhood socioeconomic position and later drug use [15], few have examined drug-related disorders or problematic opioid or polydrug use, and follow-ups were typically limited. A more recent prospective population-based study from Sweden shows that adolescent poverty is associated with increased risk of later substance use disorders [16]. In Finland, premature alcohol-attributable mortality and adolescent hospital-presenting morbidity due to psychoactive substance use disproportionately affect those from lower socioeconomic backgrounds [17, 18] and single-parent households [18].

Despite the importance of considering socioeconomic antecedents of later drug-related mortality, prospective population-wide studies remain scarce. Most existing evidence stems from retrospective or survey-based designs, which are subject to selective non-response, preferential reporting, and attrition, or are based on selected subpopulations, which may suffer from low generalizability. To our knowledge, only one study has estimated drug-related mortality risks by childhood social characteristics at the population level. Gauffin et al. [19] found that lower childhood socioeconomic position was associated with higher risks of drug-related deaths, hospital admissions, and crime between ages 16 and 35. However, their follow-up ended in 2008. Given the recent increases in drug-induced deaths [5–8] and the prevalence of drug use disorders [3], analyses of more recent birth cohorts are needed to better understand how drug use contributes to current socioeconomic disparities in mortality.

This study investigates whether social characteristics in childhood predict subsequent drug-related mortality. We utilize full-population data from high-quality Finnish administrative registers to estimate drug-related mortality risks at ages 16–41 by parental education, household income, and household type at age 15. We evaluate the independent net associations of these factors, as well as assess whether their co-occurrence poses a particularly high risk for drug-related mortality.

## Methods

### Data

We utilized nation-wide administrative register data on annual sociodemographic and causes of death information maintained, pseudonymized and approved for research (TK-53-1490-18) by Statistics Finland.

### Study population

**Figure 1.**
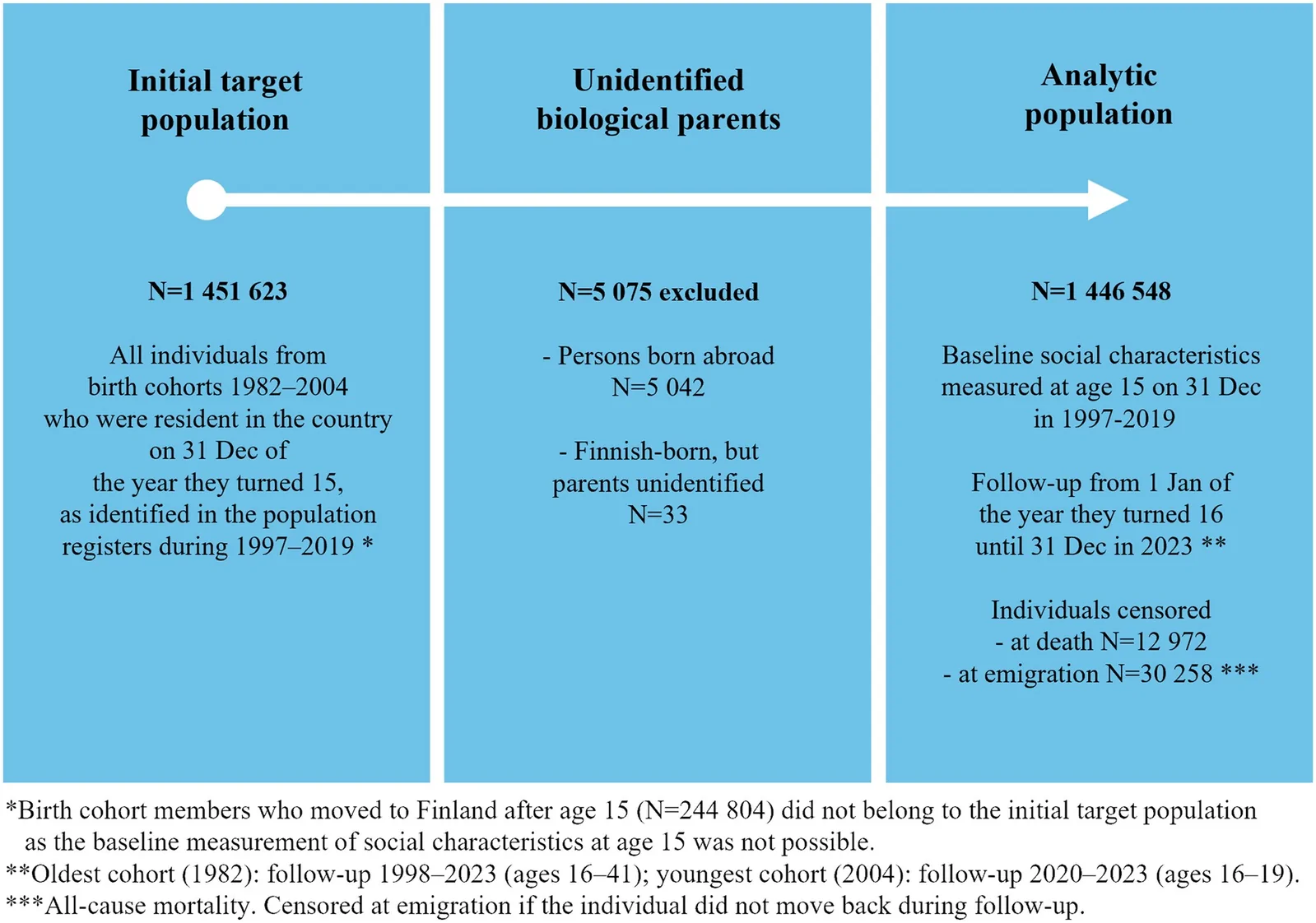
Flow chart of the selection of the analytic population (*N* = 1 446 548).

We identified all members of birth cohorts 1982–2004 present in the population in 1997–2019, when they were 15 years old. We excluded those who did not have linkages to either biological parent (Fig. 1), resulting in analytic population of *N* = 1 446 548 individuals.

### Outcome variable

We identified those who died of drug-related causes from the Causes of Death Register. To ensure a comprehensive assessment of drug-related mortality in Finland, deaths were included if the underlying cause was drug poisoning or if the underlying or one of the contributory causes was a drug-attributable mental or behavioural disorder. The underlying cause of death recorded on the death certificate refers to the condition that initiated the process that led to death. Contributing causes of death may be included in the death certificate when an illness or injury is considered to have influenced the development of the underlying cause or significantly worsened the individual’s overall condition [20]. Among individuals with high-risk drug use history, mortality due to other underlying causes than poisonings is also elevated. For example, deaths due to infections, accidents, suicides and homicides are closely intertwined with problematic drug use [21]. Our selection of diagnostic codes followed EUDA’s definition of drug-induced deaths [4], but whereas the EUDA definition includes only deaths in which the underlying cause is drug-related, we extended the same drug-related disorder codes to contributory causes. The diagnostic codes from the International Classification of Diseases (ICD) 10th revision are detailed in Supplementary Appendices, Table A1, and the distribution of the underlying causes of the drug-related deaths in Supplementary Table A2.

### Social characteristics

We included three social characteristics as predictors: parental education, household income, and household type. Parental education was defined as the highest education attained by either biological parent. We considered three categories: basic or unknown, secondary, and tertiary. Household income was formed by dividing the sum of disposable income of all household members by the number of household consumption units. Households were divided into income deciles in the total population, which were recategorized into quintiles and tertiles. Those with missing household income (*N* = 19 267), which includes children living in institutions or without a permanent address [22], were further categorized as ‘not in a household’. We considered four categories of household type according to the child’s living situation: nuclear family (both biological parents), single-parent family (one biological parent), blended family (one biological parent and the parent’s partner), and other (neither biological parent) to capture differences in family structure and household resources.

### Covariates

All models were adjusted by a measure of urbanicity due to observed geographical differences in healthcare and substance use-related services [23] and drug-related mortality [24]. This seven-category measure by the Finnish Environmental Institute ranges from the most urban to the most rural area [25]. All models were also adjusted for the child’s birth year to account for birth cohort differences and temporal changes in the drug markets and the social environment. Analyses were conducted separately by sex, except for the interaction models where sex was included as a covariate.

Social characteristics and other covariates were measured before the start of follow-up on 31 December of the year during which the individual turned 15.

### Statistical analysis

Cox proportional hazards regression models were used to estimate differences in drug-related mortality by childhood social characteristics. The analytic population was followed from 1 January of the year in which participants turned 16 until the date of death, emigration, or the end of the study period (31 December 2023), whichever occurred first. By the end of the study period, participants were 19–41 years old. These estimates are reported as hazard ratios (HRs) with 95% confidence intervals (CIs). For descriptive purposes, we calculated crude incidence rates (IR) of drug-related deaths with 95% CIs using a Poisson model.

We first estimated the associations by each predictor separately (Models 1–3) and then with all three in the same model (Model 4). These main effects models were analysed separately for men and women. We then estimated the hazards for co-occurring predictors by interactions between parental education and household income, and between parental education and household type. These interaction models were assessed for men and women together, adjusting for sex, and with recategorized predictors to gain more statistical power. ‘Basic or unknown’ and ‘secondary’ parental education were combined, and income deciles were recategorized into three groups (lowest income: deciles 1–3; middle income: deciles 4–7; highest income: deciles 8–10). As the cross-tabulated incidence rates (Supplementary Table A3) were similar for children living with one biological parent (i.e. single-parent and blended families), these categories were analysed collectively in the interaction models. The interaction models were adjusted for the third predictor not involved in the interaction (household income/household type). The interaction with income excludes the children not living in a household (*N* = 19 267) as that category reflects household type rather than income.

Kaplan-Meier curves were plotted to assess the proportional hazards assumption (Supplementary Figs A1–A3). We also conducted tests of proportionality by modelling Schoenfeld residuals. Initial tests suggested evidence of non-proportionality by sex. We compared cohorts with Kaplan-Meier curves as a sensitivity analysis (Supplementary Figs A4–A6) to see if the more recent cohorts were more affected by the increased drug-induced mortality [7], and whether there were notable differences in the associations between social characteristics and drug-related mortality. The analytic population was divided into cohorts, with five earliest consecutive birth years, 1982–1986, considered together, thus forming our first analytic cohort with the longest follow-up time. Six consecutive birth years formed the later cohorts: 1987–1992, 1993–1998, and 1999–2004.

Our selection of diagnostic codes followed EUDA’s definition of drug-induced deaths [4], but we also included deaths with a drug-related disorder listed as a contributory cause. For more internationally comparable results, we also provided results for the models 1–4 using EUDA’s definition of drug-induced death, which identifies deaths only according to the underlying cause (Supplementary Table A4). Finally, we analysed non-drug-related mortality as a competing risk for drug-related mortality using the Fine & Gray model (Supplementary Table A5). All analyses were performed using STATA V.18.

## Results

Drug-related mortality rates were over 3× higher among men than women (Table 1; men: IR: 21.9, 95% CI: 21.1–22.8; women: IR: 5.7, 95% CI: 5.3–6.2 per 100 000 person-years). Among both men and women, the incidence gradually increased with decreasing household income. The incidence rate differences between the highest and lowest income quintiles were 17.4 (men) and 5.6 (women). A similarly graded pattern was observed by parental education, with the highest rate among those with basic or unknown parental education. The rate differences between those with basic or unknown parental education and those with tertiary parental education were 27.5 (men) and 7.0 (women). The rates were very similar among those living in single-parent and blended families and elevated compared to men and women from nuclear families. The rates were highest among those not living with either biological parent, 99.3, 95% CI: 86.9–113.5 (men) and 32.2, 95% CI: 25.4–40.9 (women).

**Table 1.** Study population, person-years at risk (/100 000), drug-related deaths and incidence rates (drug-related deaths/100 000 person-years at risk) with 95% confidence intervals, for men and women by household income quintile, highest parental education, and household type

Men	Population	Person-years		Drug-related deaths	Incidence rate	
	*N*	PY/100 000	% of PY	*D*	D/100 000 PY	95% CI
Household income, quintiles						
5th (highest)	100 194	14.9	13.4	197	13.2	[11.5–15.2]
4th	137 501	20.9	18.7	302	14.5	[12.9–16.2]
3rd	170 708	25.9	23.3	488	18.8	[17.2–20.6]
2nd	172 225	25.8	23.2	612	23.7	[21.9–25.7]
1st	148 717	22.6	20.3	691	30.6	[28.4–33.0]
Not in a household*	10 152	1.3	1.1	149	116.4	[99.1–136.6]
Highest parental education						
Tertiary	388 996	56.1	50.4	822	14.7	[13.7–15.7]
Secondary	294 582	45.9	41.2	1 222	26.6	[25.2–28.2]
Basic or unknown	55 919	9.4	8.4	395	42.1	[38.2–46.5]
Household type						
Nuclear family	482 960	74.2	66.7	984	13.3	[12.5–14.1]
Single parent	160 147	23.4	21.0	833	35.6	[33.3–38.1]
Blended family	80 518	11.6	10.4	406	35.1	[31.9–38.7]
Other	15 872	2.2	2.0	216	99.3	[86.9–113.5]
Total	739 497	111.4	100.0	2 439	21.9	[21.1–22.8]

Women	Population	Person-years		Drug-related deaths	Incidence rate	
	*N*	PY/100 000	% of PY	*D*	D/100 000 PY	95% CI
Household income, quintiles						
5th (highest)	96 161	14.1	13.3	38	2.7	[2.0–3.7]
4th	129 480	19.5	18.4	89	4.6	[3.7–5.6]
3rd	162 595	24.7	23.3	110	4.5	[3.7–5.4]
2nd	165 522	24.8	23.3	135	5.4	[4.6–6.4]
1st	144 178	21.9	20.6	182	8.3	[7.2–9.6]
Not in a household*	9 115	1.2	1.1	50	43.4	[32.9–57.2]
Highest parental education						
Tertiary	371 501	53.3	50.2	190	3.6	[3.1–4.1]
Secondary	282 244	43.9	41.3	319	7.3	[6.5–8.1]
Basic or unknown	53 306	9.0	8.4	95	10.6	[8.7–13.0]
Household type						
Nuclear family	458 912	70.2	66.1	191	2.7	[2.4–3.1]
Single parent	155 001	22.6	21.3	236	10.4	[9.2–11.8]
Blended family	78 006	11.2	10.6	109	9.7	[8.1–11.7]
Other	15 132	2.1	2.0	68	32.2	[25.4–40.9]
Total	707 051	106.2	100.0	604	5.7	[5.3–6.2]

Abbreviations: CI, confidence interval; D, deaths; PY, person-years. *Persons not identified in household-dwelling units in the register data according to the Population Information System of the Digital and Population Data Services Agency.

The Cox regression analyses for men showed increasing hazards with decreasing household income and parental education (Table 2, Models 1–2). Men in the lowest income quintile and education category had over twice the hazards (HR: 2.57, 95% CI: 2.19–3.01; HR: 2.64, 95% CI: 2.33–2.98, respectively) compared to those with the highest respective socioeconomic indicators. Individuals identified as not in households had over six-fold hazards (HR: 6.03, 95% CI: 3.92–9.29) compared to the highest income group. The results for household type (Model 3) indicate increased hazards for all categories compared to nuclear families, with HRs ranging between 2.55 (95% CI: 2.32–2.80) for single-parent households and 5.55 (95% CI: 4.47–6.89) among those not residing with a biological parent. The same patterns were generally observed for women (Table 2), although the point estimates for lower household income and non-nuclear families were somewhat higher, and CIs were wider across all predictors due to fewer drug-related deaths. For instance, the hazards were over three-fold among women in the lowest income quintile (HR: 3.48, 95% CI: 2.45–4.94), and women from single-parent households (HR: 3.51, 95% CI: 2.89–4.27).

**Table 2.** Hazard ratios for drug-related mortality for men and women by household income quintile, highest parental education, and household type with 95% confidence intervals

Men	Model 1		Model 2		Model 3		Model 4	
	HR	95% CI	HR	95% CI	HR	95% CI	HR	95% CI
Household income, quintiles								
5th (highest) (ref.)	1.00						1.00	
4th	1.15	[0.96–1.38]					1.03	[0.86–1.23]
3rd	1.56	[1.32–1.85]					1.24	[1.05–1.47]
2nd	2.01	[1.71–2.36]					1.32	[1.11–1.56]
1st	2.57	[2.19–3.01]					1.41	[1.18–1.67]
Not in a household*	6.03	[3.92–9.29]					1.32	[0.80–2.16]
Highest parental education								
Tertiary (ref.)			1.00				1.00	
Secondary			1.92	[1.76–2.10]			1.56	[1.42–1.71]
Basic or unknown			2.64	[2.33–2.98]			1.86	[1.63–2.11]
Household type								
Nuclear family (ref.)					1.00		1.00	
Single parent					2.55	[2.32–2.80]	2.10	[1.89–2.33]
Blended family					2.63	[2.34–2.95]	2.34	[2.08–2.63]
Other					5.55	[4.47–6.89]	4.15	[3.22–5.35]

Women	Model 1		Model 2		Model 3		Model 4	
	HR	95% CI	HR	95% CI	HR	95% CI	HR	95% CI
Household income, quintiles								
5th (highest) (ref.)	1.00						1.00	
4th	1.83	[1.25–2.68]					1.58	[1.08–2.32]
3rd	1.88	[1.30–2.72]					1.37	[0.94–2.00]
2nd	2.31	[1.61–3.32]					1.28	[0.88–1.87]
1st	3.48	[2.45–4.94]					1.53	[1.05–2.24]
Not in a household*	14.29	[7.11–28.72]					2.85	[1.24–6.55]
Highest parental education								
Tertiary (ref.)			1.00				1.00	
Secondary			2.20	[1.83–2.64]			1.73	[1.43–2.09]
Basic or unknown			2.68	[2.08–3.46]			1.78	[1.37–2.32]
Household type								
Nuclear family (ref.)					1.00		1.00	
Single parent					3.51	[2.89–4.27]	3.06	[2.46–3.79]
Blended family					3.46	[2.73–4.38]	3.09	[2.44–3.93]
Other					8.08	[5.46–11.95]	5.15	[3.14–8.44]

Results are from Cox regression models. Models 1–3 are main effects models with one predictor. Model 4 is mutually adjusted for all three predictors. All models are adjusted by urbanicity and birth year.

Abbreviations: CI, confidence interval; HR, hazard ratio. *Persons not identified in household-dwelling units in the register data according to the Population Information System of the Digital and Population Data Services Agency.

In the mutually adjusted models, the associations attenuated for both men and women (Table 2, Model 4). Although the graded associations for income and education mostly persisted, estimates for household income were small after adjustment for education and household type, and some CIs included one. Lower parental education remained associated with drug-related mortality after adjustment for income and household type, with increased hazards among those with secondary, and basic or missing parental education. The largest associations were retained for household type; all HRs remained above 2.00 for men and above 3.00 for women residing in non-nuclear families after adjusting for education and income.

**Figure 2.**
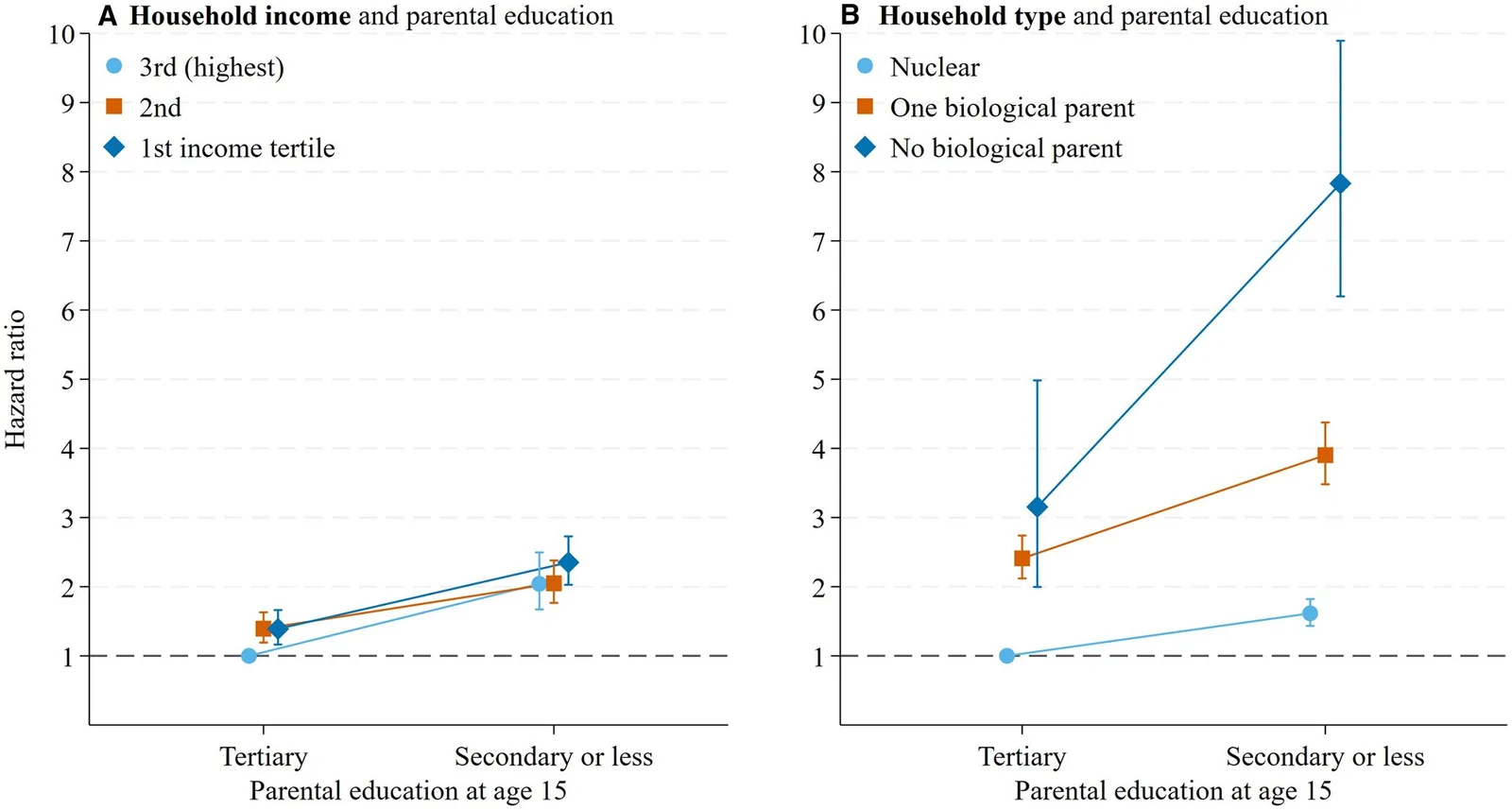
Hazard ratios of the interaction between household income and parental education (A) and household type and parental education (B) for drug-related mortality with 95% confidence intervals for combined men and women. The Cox regression models were adjusted for the household type (A)/household income (B), year of birth, sex and urbanicity. In the first interaction model (A) children outside of dwelling population were excluded (Household income: ‘Not in a household’). Reference category (A): at least one biological parent with tertiary education in a household belonging to the third (highest) income group. Reference category (B): at least one parent with tertiary education and child lives with both biological parents (nuclear family) (Log scale in Supplementary Appendices, Fig. A7).

The interaction between household income and parental education (Fig. 2A) showed that income groups were similar within each educational group. The interaction between household type and parental education (Fig. 2B) indicated larger hazards by household type among those with lower-educated parents relative to those with tertiary-educated parents, though with large CIs in the groups containing individuals living without a biological parent. Compared to those living in nuclear families with at least one parent with tertiary education, the hazards were over twice as high among children with at least one tertiary-educated parent who were living with one biological parent (HR: 2.41, 95% CI: 2.12–2.74) and more than 3× as high for those living without a biological parent (HR: 3.15, 95% CI: 2.00–4.98). Among children with lower parental education, the hazards increased as the number of co-resident biological parents decreased (nuclear family, HR: 1.62, 95% CI: 1.43–1.82; one biological parent, HR 3.90, 95% CI: 3.48–4.37; no biological parents, HR 7.83, 95% CI: 6.20–9.89).

A cohort comparison showed that those born in 1999–2004 (followed for 4 to 9 years) were more likely to die from drug-related causes at ages 16–24 than the other cohorts (Supplementary Fig. A4). These cohort differences were larger among those with lower-educated parents, low-income families and households with neither biological parent (Supplementary Figs A4–A6). There were no cohort differences among men and women from nuclear households.

A sensitivity analysis was conducted for models 1–4 using EUDA’s definition of drug-induced death [4]. These estimates (Supplementary Table A4) were similar to those presented in Table 2 but with wider CIs given the smaller number of included events. A second set of sensitivity analyses was conducted for models 1–4 using drug-related deaths as the outcome and non-drug-related deaths as the competing risk. These estimates (Supplementary Table A5) were highly similar to the main analyses presented in Table 2.

## Discussion

Our study extends the literature by using total population Finnish register data to estimate independent and co-occurring associations between household income, parental education, and household type at age 15 and drug-related mortality between ages 16 and 41. Among both men and women, we found that the hazards for drug-related mortality were consistently higher among those from families with lower income and parental education, and who did not reside with both biological parents. The independent associations between drug-related mortality and parental education and household type remained robust to mutual adjustment, and the interaction analyses further suggested that the co-occurrence of lower parental education and non-nuclear household type was associated with larger relative increases in drug-related mortality. These mortality differences were observed consistently among birth cohorts despite differences in follow-up time, suggesting that the association has not diminished.

Those with lower-educated parents or from low-income households had a more than two-fold risk of drug-related death compared to their counterparts with tertiary-educated parents or from high-income households, with a stepwise increase in risk with decreasing parental education and household income levels. The associations were still observed after mutual adjustment of the other social characteristics, albeit attenuated, and the graded associations mostly remained. These graded associations of parental education and income are in line with the results from Sweden, where those from the lowest childhood socioeconomic stratum had a two-fold risk of drug-related death, hospital admissions, and criminality compared to those from the highest stratum, with decreased risks with increasing socioeconomic position thereafter [19]. Such a gradient was not observed in the only prior Finnish population-wide study [13]. However, that study used attained socioeconomic position and was therefore potentially limited by reverse causation.

Household type was a particularly salient predictor of later drug-related death. Living in single-parent and blended households at age 15 was associated with an elevated risk of drug-related mortality compared to those living with both parents, even after controlling for parental education and household income. Previous studies have indicated that stressful events, such as parental separation, are associated with an increased risk of substance use-attributable morbidity and mortality [26, 27]. Similarly, underlying factors, including parental substance use or mental health problems, are associated with an elevated risk of union dissolution among parents [28, 29] as well as an increased risk of substance use among children [30].

The highest risks were observed among individuals not living with either biological parent. However, this group is heterogeneous, including children placed in out-of-home care in foster-families and child welfare institutions [31]. Out-of-home placement is often associated with parental mental health or substance misuse [32] which likely contributes to the elevated risk of drug use in this subpopulation, and is also observed in other studies [33]. Additionally, the child’s own mental health and substance use problems may be related to placement in out-of-home care [32]. In addition to selection, other mechanisms, such as adverse peer influence within institutions, could also play a role [34, 35].

We found limited evidence that household income was independently or cumulatively associated with drug-related mortality. Conversely, the independent and cumulative risks captured by parental education and household type indicate that immaterial resources, such as parental influence on health behaviours and social resources [36], may play a more significant protective role than material resources. However, unobserved familial factors, including genetic predispositions and adoption of health risk behaviours, may have confounded the observed associations to some extent [37, 38]. Stressful events, such as parental separation, may also trigger genetic predispositions [39]. Further research incorporating sibling or cousin comparisons to account for unmeasured familial confounding may yield deeper insights into these associations.

The increased quantity and variety of substances available in European drug markets, and the unknown harms related to the use of new and potent synthetic drugs and their concurrent use with other substances, pose further risks for drug-related mortality among young people [2]. The childhood socioeconomic gradient in drug-related deaths highlights a life course phenomenon rooted in early life social conditions, not only in proximal adult behaviours. As such, targeted interventions and access to services already in adolescence could especially benefit those from families with lower socioeconomic positions.

### Strengths and limitations

Our study used high-quality register data for 1.4 million Finnish residents born in 1982–2004. Finnish cause-of-death data have high validity for identifying drug-related deaths: medicolegal investigations into unexpected or unclear deaths are stipulated by law and were carried out in around 90% of such deaths in 2000–2003 [40]. By measuring family background characteristics at age 15, we could account for socioeconomic antecedents of later drug use, thus avoiding reverse causation problems present in prior research. As existing Nordic population-based evidence was based on data until 2008 [13, 19], our extensive follow-up period and large number of cohorts highlight the persistence of socioeconomic differences in premature drug-related mortality. Given the relative generosity of the Finnish welfare system, the estimated hazard ratios may be higher in settings with less comprehensive social security.

Our research focus necessitated having an identifiable link to at least one biological parent and being in the population at age 15 to measure childhood social characteristics. Only 0.3% of the original target population was excluded due to missing biological parent linkages, which is unlikely to affect our main results. Among individuals living with neither biological parent, reverse causality is possible as the child’s own substance use problem may be related to their out-of-home placement. However, other mechanisms, such as parental mental health and substance use problems, or adverse peer influence in institutions may play a role. This heterogeneous group of children constituted 2.1% of the analytic population. Furthermore, while fixing the measurement of family background at age 15 simplifies the analyses, it does not consider the whole childhood context. The role of changes in family characteristics occurring at potentially vulnerable child or adolescent ages deserve further research. However, we believe our choice to focus on family background at age 15 is an appropriate approximation of the childhood family background on the population level.

We defined drug-related deaths based on both underlying and contributing causes of death. Sensitivity analyses that only included the underlying cause of death produced similar results but with wider CIs (Supplementary Table A4), arising from the rarity of the event. Mortality from other underlying causes, including suicides, accidents and homicide, is elevated among individuals with problematic drug use [21]. Therefore, including also contributory causes of drug-related deaths offers a more comprehensive understanding of drug mortality.

## Conclusions

Individuals from non-nuclear families, especially those whose parents have lower educational attainment, may face elevated risks of drug-related mortality. These observed disparities are likely influenced by multiple mechanisms, including social causation explanations and family-level confounding. Although drug-related deaths remain relatively rare in Finland, they are a significant cause of premature death among the young, and this increasing trend [7] could have broader implications for health inequalities in the coming years.

## Supplementary Material

ckag083_Supplementary_Data

## Data Availability

Due to data protection regulations of the national register-holders, we are unable to make any parts of the data available to third parties. Interested researchers may contact Statistics Finland (http://www.stat.fi/tup/mikroaineistot/index_en.html). Key points Lower parental socioeconomic position and not living with both parents in childhood predict an increased risk of drug-related mortality. Highest risk among children of lower educated parents from non-nuclear families. Differences in drug-related deaths persist across birth cohorts 1982–2004. Identifying early life social- and family-related risk factors may inform preventative measures to reduce drug-related deaths.
