# Supplementary material for: Childhood social characteristics and drug-related mortality by age 41: a register-based study of Finnish birth cohorts 1982–2004
Source: Eur J Public Health. 2026 Jun 16;36(4):ckag083. doi: 10.1093/eurpub/ckag083 (PMC13271240; doi:10.1093/eurpub/ckag083)
Supplement: ckag083_Supplementary_Data [file ckag083_supplementary_data.docx]

# Appendices

**Table A1** The selection of diagnostic codes* based on the WHO International Classification of Diseases (ICD) 10^th^ revision. The death was considered as drug-related if the underlying cause of death was drug poisoning or if the underlying or one of the contributory causes was a mental or behavioural disorder due to drug use.

| **Diagnosis** | **ICD-10** |
| --- | --- |
| *As the underlying or contributory cause of death* |  |
| **Mental and behavioural disorders (incl. acute intoxication, harmful use, dependence, and other mental and behavioural disorders) due to use of:** |  |
| Opioids | F11 |
| Cannabinoids | F12 |
| Cocaine | F14 |
| Stimulants | F15 |
| Hallucinogens | F16 |
| Multiple drug use | F19 |
|  |  |
| *As the underlying cause of death in combination with the substance code(s)* |  |
| **External causes of poisonings:** |  |
| Accidental poisoning | X42 (^1^), X41 (^2^) |
| Intentional poisoning | X62 (^1^), X61 (^2^) |
| Poisoning, undetermined intent | Y12 (^1^), Y11 (^2^) |
| Exposure to other and unspecified drugs (accidental, intentional, undetermined intent) | X44(^3^), X64(^3^), Y14(^3^) |
|  |  |
| (1) In combination with T-codes: T40.0–40.9. |  |
| (2) In combination with T-code: T43.6. |  |
| (3) In combination with T codes: T40.0-T40.9 or T43.6. |  |
|  |  |

*Our selection followed EUDA’s definition of drug-induced deaths (1), but whereas the EUDA definition includes only deaths in which the underlying cause is drug-related, we extended the same drug-related disorder codes to contributory causes. For more internationally comparable results, we also provided results for the models 1–4 using EUDA’s definition of drug-induced death (Table A4).

**Table A2** The distribution of the underlying causes of the drug-related deaths among the analytic population.

| **Underlying causes of death when the death was drug-induced (ICD-10)** | **N** | **%** |
| --- | --- | --- |
| Accidental poisoning by and exposure to drugs (narcotics, psychodysleptics) (X42) | 1 118 | 66.1 |
| Mental and behavioural disorders due to use of multiple drug use and use of other psychoactive substances (F19) | 246 | 14.6 |
| Accidental poisoning by and exposure to drugs (psychotropic medicines) (X41) | 152 | 9.0 |
| Intentional self-poisoning by and exposure to drugs (narcotics, psychodysleptics) (X62) | 97 | 5.7 |
| Poisoning by and exposure to drugs, undetermined intent (narcotics, psychodysleptics) (Y12) | 38 | 2.3 |
| Mental and behavioural disorders due to use of opioids (F11) | 16 | 1.0 |
| Poisoning by and exposure to drugs, undetermined intent (psychotropic medicines) (Y11) | 7 | 0.4 |
| Mental and behavioural disorders due to use of stimulants (F15) | 7 | 0.4 |
| Intentional self-poisoning by and exposure to drugs (psychotropic medicines) (X61) | 5 | 0.3 |
| Other (mental and behavioural disorders due to use of cannabinoids (F12), hallucinogens (F16) or accidental (X44) or intentional (X64) self-poisoning by and exposure to drugs (other and unspecified drugs, medicaments and biological substance)) ** | 5 | 0.3 |
| *Total* | *1 691* | *100.0* |
| **Underlying causes of death* when one of the contributing causes was drug-related (F11, F12, F14, F15, F16, F19)** | **N** | **%** |
| Suicides (X60-X84, Y870) | 566 | 41.9 |
| Land traffic accidents | 124 | 9.2 |
| Accidental poisonings excl. accidental poisoning by alcohol (X40-X44, X46-X49, Y10-Y15) | 103 | 7.6 |
| Other accidents and sequelae of accidents | 86 | 6.4 |
| Assault (X85-Y09, Y871) | 86 | 6.4 |
| Other heart diseases excl. rheumatic and alcohol-related (I30-I425, I427-I52) | 75 | 5.5 |
| Event of undetermined intent (Y16-Y34, Y872) | 66 | 4.9 |
| Diabetes mellitus (E10-E14) | 39 | 2.9 |
| Alcohol-related diseases and accidental poisoning by alcohol | 32 | 2.4 |
| Accidental drownings (W65-W74) | 29 | 2.1 |
| Accidental falls (W00-W19) | 28 | 2.1 |
| Other diseases of the circulatory system (I00-I15, I26-I28, I70-I99) | 18 | 1.3 |
| Cerebrovascular diseases (I60-I69) | 12 | 0.9 |
| Other diseases of the nervous system and sense organs excl. alcohol-related | 11 | 0.8 |
| Pneumonia (J12-J18, J849) | 10 | 0.7 |
| Diseases of the digestive system excl. alcohol-related diseases | 10 | 0.7 |
| Water transport accidents (V90-V94) | 9 | 0.7 |
| Other land transport accidents | 8 | 0.6 |
| Other diseases excl. alcohol-related | 7 | 0.5 |
| Other infectious and parasitic diseases (A00-A09, A20-B19, B25-B89, B91-B99) | 6 | 0.4 |
| Ischaemic heart diseases (I20-I25) | 6 | 0.4 |
| Other ** | 21 | 1.6 |
| *Total* | *1 352* | *100.0* |
| * Classification based on Causes of death, national time series classification 2021 (2). |  |  |
| ** Causes of N<5 not shown. |  |  |

**Table A3** Cross-tabulated study population (N), follow-up time (PY; person-years at risk), drug-related deaths (D), incidence rates (IR; drug-related deaths/100 000 person-years at risk) by highest parental education and household income or household type for men and women.

| **Men** | *Highest parental education:* Tertiary | | | |  | *Highest parental education:* Secondary | | | | | *Highest parental education:* Basic or unknown | | | | |
| --- | --- | --- | --- | --- | --- | --- | --- | --- | --- | --- | --- | --- | --- | --- | --- |
|  | **N** | **PY/100 000** | **D** | **IR** | **95% CI** | **N** | **PY/100 000** | **D** | **IR** | **95% CI** | **N** | **PY/100 000** | **D** | **IR** | **95% CI** |
| *Household income, quintiles* | |  |  |  |  |  |  |  |  |  |  |  |  |  |  |
| 5th (highest) | 83 244 | 12.1 | 134 | 11.1 | [9.3–13.1] | 14 516 | 2.3 | 47 | 20.1 | [15.1–26.8] | 2 434 | 0.4 | 16 | 36.0 | [22.1–58.8] |
| 4th | 94 339 | 13.7 | 136 | 9.9 | [8.4–11.7] | 38 040 | 6.2 | 138 | 22.3 | [18.9–26.4] | 5 122 | 1.0 | 28 | 29.0 | [20.0–42.0] |
| 3rd | 91 981 | 13.2 | 221 | 16.7 | [14.6–19.0] | 70 128 | 11.1 | 219 | 19.7 | [17.3–22.5] | 8 599 | 1.6 | 48 | 30.4 | [22.9–40.4] |
| 2nd | 72 151 | 10.2 | 195 | 19.1 | [16.6–21.9] | 86 821 | 13.2 | 324 | 24.5 | [21.9–27.3] | 13 253 | 2.3 | 93 | 40.4 | [33.0–49.5] |
| 1st | 45 158 | 6.5 | 121 | 18.5 | [15.5–22.1] | 80 300 | 12.4 | 425 | 34.3 | [31.2–37.7] | 23 259 | 3.7 | 145 | 39.6 | [33.7–46.7] |
| Not in a household | 2 123 | 0.2 | 15 | 61.7 | [37.2–102.4] | 4 777 | 0.6 | 69 | 113.6 | [89.7–143.8] | 3 252 | 0.4 | 65 | 151.2 | [118.5–192.8] |
|  |  |  |  |  |  |  |  |  |  |  |  |  |  |  |  |
| *Household type* |  |  |  |  |  |  |  |  |  |  |  |  |  |  |  |
| Nuclear family | 282 541 | 41.3 | 429 | 10.4 | [9.4–11.4] | 175 754 | 28.4 | 470 | 16.5 | [15.1–18.1] | 24 665 | 4.4 | 85 | 19.1 | [15.5–23.7] |
| Single parent | 70 466 | 9.8 | 254 | 25.8 | [22.9–29.2] | 72 332 | 10.7 | 437 | 40.7 | [37.0–44.7] | 17 349 | 2.8 | 142 | 50.4 | [42.8–59.4] |
| Blended family | 32 897 | 4.5 | 121 | 26.7 | [22.3–31.9] | 39 368 | 5.7 | 219 | 38.3 | [33.5–43.7] | 8 253 | 1.3 | 66 | 50.7 | [39.8–64.5] |
| Other | 3 092 | 0.4 | 18 | 46.5 | [29.3–73.7] | 7 128 | 1.0 | 96 | 98.4 | [80.5–120.2] | 5 652 | 0.8 | 102 | 125.7 | [103.5–152.7] |
|  |  |  |  |  |  |  |  |  |  |  |  |  |  |  |  |
| *Total* | 388 996 | 56.1 | 822 | 14.7 | [13.7–15.7] | 294 582 | 45.9 | 1 222 | 26.6 | [25.2–28.2] | 55 919 | 9.4 | 395 | 42.1 | [38.2–46.5] |
|  | | | | | | | | | | | | | | | |
| **Women** | *Highest parental education:* Tertiary | | | |  | *Highest parental education:* Secondary | | | | | *Highest parental education:* Basic or unknown | | | | |
|  | **N** | **PY/100 000** | **D** | **IR** | **95% CI** | **N** | **PY/100 000** | **D** | **IR** | **95% CI** | **N** | **PY/100 000** | **D** | **IR** | **95% CI** |
| *Household income, quintiles* | |  |  |  |  |  |  |  |  |  |  |  |  |  |  |
| 5th (highest) | 79 838 | 11.5 | 24 | 2.1 | [1.4–3.1] | 14 086 | 2.2 | 14 | 6.2 | [3.7–10.5] | 2 237 | 0.4 | 0 | 0.0 | - |
| 4th | 89 035 | 12.9 | 37 | 2.9 | [2.1–4.0] | 35 722 | 5.8 | 42 | 7.3 | [5.4–9.9] | 4 723 | 0.9 | 10 | 11.2 | [6.0–20.9] |
| 3rd | 87 677 | 12.6 | 42 | 3.3 | [2.5–4.5] | 66 532 | 10.5 | 55 | 5.2 | [4.0–6.8] | 8 386 | 1.5 | 13 | 8.4 | [4.9–14.5] |
| 2nd | 69 484 | 9.9 | 45 | 4.6 | [3.4–6.1] | 83 369 | 12.7 | 71 | 5.6 | [4.4–7.0] | 12 669 | 2.2 | 19 | 8.7 | [5.5–13.6] |
| 1st | 43 550 | 6.3 | 37 | 5.9 | [4.3–8.1] | 78 250 | 12.1 | 105 | 8.7 | [7.2–10.5] | 22 378 | 3.5 | 40 | 11.3 | [8.3–15.5] |
| Not in a household | 1 917 | 0.2 | 5 | 22.9 | [9.5–54.9] | 4 285 | 0.5 | 32 | 59.2 | [41.8–83.6] | 2 913 | 0.4 | 13 | 33.1 | [19.2–57.0] |
|  |  |  |  |  |  |  |  |  |  |  |  |  |  |  |  |
| *Household type* |  |  |  |  |  |  |  |  |  |  |  |  |  |  |  |
| Nuclear family | 268 469 | 39.1 | 86 | 2.2 | [1.8–2.7] | 167 324 | 27.0 | 80 | 3.0 | [2.4–3.7] | 23 119 | 4.2 | 25 | 6.0 | [4.1–8.9] |
| Single parent | 68 259 | 9.5 | 70 | 7.4 | [5.8–9.3] | 69 925 | 10.4 | 131 | 12.6 | [10.6–14.9] | 16 817 | 2.7 | 35 | 12.8 | [9.2–17.9] |
| Blended family | 31 774 | 4.4 | 29 | 6.6 | [4.6–9.5] | 38 112 | 5.5 | 69 | 12.4 | [9.8–15.7] | 8 120 | 1.3 | 11 | 8.6 | [4.8–15.5] |
| Other | 2 999 | 0.4 | 5 | 13.1 | [5.5–31.6] | 6 883 | 1.0 | 39 | 40.7 | [29.8–55.7] | 5 250 | 0.8 | 24 | 31.1 | [20.9–46.4] |
|  |  |  |  |  |  |  |  |  |  |  |  |  |  |  |  |
| *Total* | 371 501 | 53.3 | 190 | 3.6 | [3.1–4.1] | 282 244 | 43.9 | 319 | 7.3 | [6.5–8.1] | 53 306 | 9.0 | 95 | 10.6 | [8.7–13.0] |
| Abbreviations: N, study population; PY, person-years; D, drug-related deaths; IR, incidence rate; CI, confidence interval. | | | | | | | | | | | | | | | |

**Table A4** Hazard ratios for drug-induced mortality according to the underlying cause of death for men and women by household income quintile, highest parental education, and household type with 95% confidence intervals.

| **Men** | **Model 1** | | **Model 2** | | **Model 3** | | **Model 4** | |
| --- | --- | --- | --- | --- | --- | --- | --- | --- |
|  | HR | 95% CI | HR | 95% CI | HR | 95% CI | HR | 95% CI |
| *Household income, quintiles* |  |  |  |  |  |  |  |  |
| 5th (highest) (ref.) | 1.00 |  |  |  |  |  | 1.00 |  |
| 4th | 1.13 | [0.89–1.43] |  |  |  |  | 1.00 | [0.79–1.27] |
| 3rd | 1.56 | [1.25–1.94] |  |  |  |  | 1.22 | [0.97–1.52] |
| 2nd | 2.04 | [1.65–2.53] |  |  |  |  | 1.30 | [1.04–1.62] |
| 1st | 2.40 | [1.94–2.97] |  |  |  |  | 1.25 | [0.99–1.58] |
| Not in a household | 7.75 | [4.57–13.13] |  |  |  |  | 1.56 | [0.85–2.89] |
|  |  |  |  |  |  |  |  |  |
| *Highest parental education* |  |  |  |  |  |  |  |  |
| Tertiary (ref.) |  |  | 1.00 |  |  |  | 1.00 |  |
| Secondary |  |  | 1.89 | [1.68–2.14] |  |  | 1.55 | [1.36–1.76] |
| Basic or unknown |  |  | 2.89 | [2.46–3.40] |  |  | 2.06 | [1.73–2.44] |
|  |  |  |  |  |  |  |  |  |
| *Household type* |  |  |  |  |  |  |  |  |
| Nuclear family (ref.) |  |  |  |  | 1.00 |  | 1.00 |  |
| Single parent |  |  |  |  | 2.62 | [2.31–2.98] | 2.21 | [1.92–2.54] |
| Blended family |  |  |  |  | 2.59 | [2.21–3.04] | 2.29 | [1.95–2.69] |
| Other |  |  |  |  | 6.34 | [4.80–8.38] | 4.37 | [3.13–6.10] |
|  |  |  |  |  |  |  |  |  |
| **Women** | **Model 1** | | **Model 2** | | **Model 3** | | **Model 4** | |
|  | HR | 95% CI | HR | 95% CI | HR | 95% CI | HR | 95% CI |
| *Household income, quintiles* |  |  |  |  |  |  |  |  |
| 5th (highest) (ref.) | 1.00 |  |  |  |  |  | 1.00 |  |
| 4th | 1.65 | [1.00–2.71] |  |  |  |  | 1.41 | [0.86–2.32] |
| 3rd | 1.88 | [1.17–3.03] |  |  |  |  | 1.35 | [0.84–2.19] |
| 2nd | 2.08 | [1.30–3.32] |  |  |  |  | 1.13 | [0.69–1.85] |
| 1st | 3.27 | [2.08–5.15] |  |  |  |  | 1.43 | [0.88–2.33] |
| Not in a household | 21.75 | [9.96–47.51] |  |  |  |  | 3.96 | [1.50–10.48] |
|  |  |  |  |  |  |  |  |  |
| *Highest parental education* |  |  |  |  |  |  |  |  |
| Tertiary (ref.) |  |  | 1.00 |  |  |  | 1.00 |  |
| Secondary |  |  | 2.34 | [1.84–2.98] |  |  | 1.79 | [1.39–2.30] |
| Basic or unknown |  |  | 2.73 | [1.94–3.83] |  |  | 1.73 | [1.21–2.46] |
|  |  |  |  |  |  |  |  |  |
| *Household type* |  |  |  |  |  |  |  |  |
| Nuclear family (ref.) |  |  |  |  | 1.00 |  | 1.00 |  |
| Single parent |  |  |  |  | 3.73 | [2.86–4.87] | 3.29 | [2.45–4.41] |
| Blended family |  |  |  |  | 4.45 | [3.29–6.03] | 3.98 | [2.93–5.41] |
| Other |  |  |  |  | 11.12 | [6.89–17.96] | 6.15 | [3.25–11.65] |
| Models 1–3 are main effects models with one predictor. Model 4 is mutually adjusted for all three predictors. | | | | | | | | |
| All models are adjusted by urbanicity and birth year. | | | | | | | | |
| Analytic population: men N=739 497; women N=707 051. | | | | | | | | |
| Drug-induced deaths as defined by EUDA (1): men D=1 346; women D=345. | | | | | | | | |
| Abbreviations: HR=hazard ratio; CI=confidence interval. | | | | | | | | |

**Table A5** Subhazard ratios for drug-related deaths with non-drug-related deaths as the competing risk for men and women by household income quintile, highest parental education, and household type with 95% confidence intervals. Results from Fine & Gray models.

| **Men** | **Model 1** | | **Model 2** | | **Model 3** | | **Model 4** | |
| --- | --- | --- | --- | --- | --- | --- | --- | --- |
|  | SHR | 95% CI | SHR | 95% CI | SHR | 95% CI | SHR | 95% CI |
| *Household income, quintiles* |  |  |  |  |  |  |  |  |
| 5th (highest) (ref.) | 1.00 |  |  |  |  |  | 1.00 |  |
| 4th | 1.13 | [0.95–1.36] |  |  |  |  | 1.02 | [0.85–1.22] |
| 3rd | 1.53 | [1.29–1.80] |  |  |  |  | 1.22 | [1.03–1.45] |
| 2nd | 1.97 | [1.68–2.32] |  |  |  |  | 1.30 | [1.10–1.54] |
| 1st | 2.55 | [2.17–2.99] |  |  |  |  | 1.40 | [1.18–1.66] |
| Not in a household | 6.13 | [4.10–9.15] |  |  |  |  | 1.33 | [0.83–2.12] |
|  |  |  |  |  |  |  |  |  |
| *Highest parental education* |  |  |  |  |  |  |  |  |
| Tertiary (ref.) |  |  | 1.00 |  |  |  | 1.00 |  |
| Secondary |  |  | 1.90 | [1.74–2.08] |  |  | 1.55 | [1.41–1.70] |
| Basic or unknown |  |  | 2.66 | [2.35–3.01] |  |  | 1.85 | [1.63–2.11] |
|  |  |  |  |  |  |  |  |  |
| *Household type* |  |  |  |  |  |  |  |  |
| Nuclear family (ref.) |  |  |  |  | 1.00 |  | 1.00 |  |
| Single parent |  |  |  |  | 2.59 | [2.36–2.85] | 2.14 | [1.93–2.37] |
| Blended family |  |  |  |  | 2.62 | [2.34–2.94] | 2.34 | [2.08–2.63] |
| Other |  |  |  |  | 5.56 | [4.49–6.88] | 4.15 | [3.23–5.34] |
|  |  |  |  |  |  |  |  |  |
| **Women** | **Model 1** | | **Model 2** | | **Model 3** | | **Model 4** | |
|  | SHR | 95% CI | SHR | 95% CI | SHR | 95% CI | SHR | 95% CI |
| *Household income, quintiles* |  |  |  |  |  |  |  |  |
| 5th (highest) (ref.) | 1.00 |  |  |  |  |  | 1.00 |  |
| 4th | 1.77 | [1.21–2.59] |  |  |  |  | 1.55 | [1.06–2.28] |
| 3rd | 1.80 | [1.24–2.61] |  |  |  |  | 1.34 | [0.92–1.96] |
| 2nd | 2.25 | [1.57–3.24] |  |  |  |  | 1.26 | [0.86–1.85] |
| 1st | 3.45 | [2.42–4.91] |  |  |  |  | 1.51 | [1.02–2.23] |
| Not in a household | 15.41 | [7.62–31.19] |  |  |  |  | 2.94 | [1.28–6.74] |
|  |  |  |  |  |  |  |  |  |
| *Highest parental education* |  |  |  |  |  |  |  |  |
| Tertiary (ref.) |  |  | 1.00 |  |  |  | 1.00 |  |
| Secondary |  |  | 2.17 | [1.81–2.61] |  |  | 1.72 | [1.41–2.08] |
| Basic or unknown |  |  | 2.76 | [2.13–3.56] |  |  | 1.80 | [1.38–2.35] |
|  |  |  |  |  |  |  |  |  |
| *Household type* |  |  |  |  |  |  |  |  |
| Nuclear family (ref.) |  |  |  |  | 1.00 |  | 1.00 |  |
| Single parent |  |  |  |  | 3.64 | [3.00–4.40] | 3.18 | [2.56–3.96] |
| Blended family |  |  |  |  | 3.48 | [2.75–4.41] | 3.12 | [2.46–3.97] |
| Other |  |  |  |  | 8.37 | [5.70–12.30] | 5.30 | [3.24–8.66] |
| Models 1–3 are main effects models with one predictor. Model 4 is mutually adjusted for all three predictors. | | | | | | | | |
| All models are adjusted by urbanicity and birth year. | | | | | | | | |
| Analytic population: men N=739 497; women N=707 051. | | | | | | | | |
| Drug-related deaths (failure event): men D=2 439; women D=604. | | | | | | | | |
| Non-drug-related deaths (competing event): men D=6 989; women D=2 940. | | | | | | | | |
| Total deaths: men D=9 428; women D=3 544. | | | | | | | | |
| Abbreviations: SHR=subhazard ratio; CI=confidence interval. | | | | | | | | |


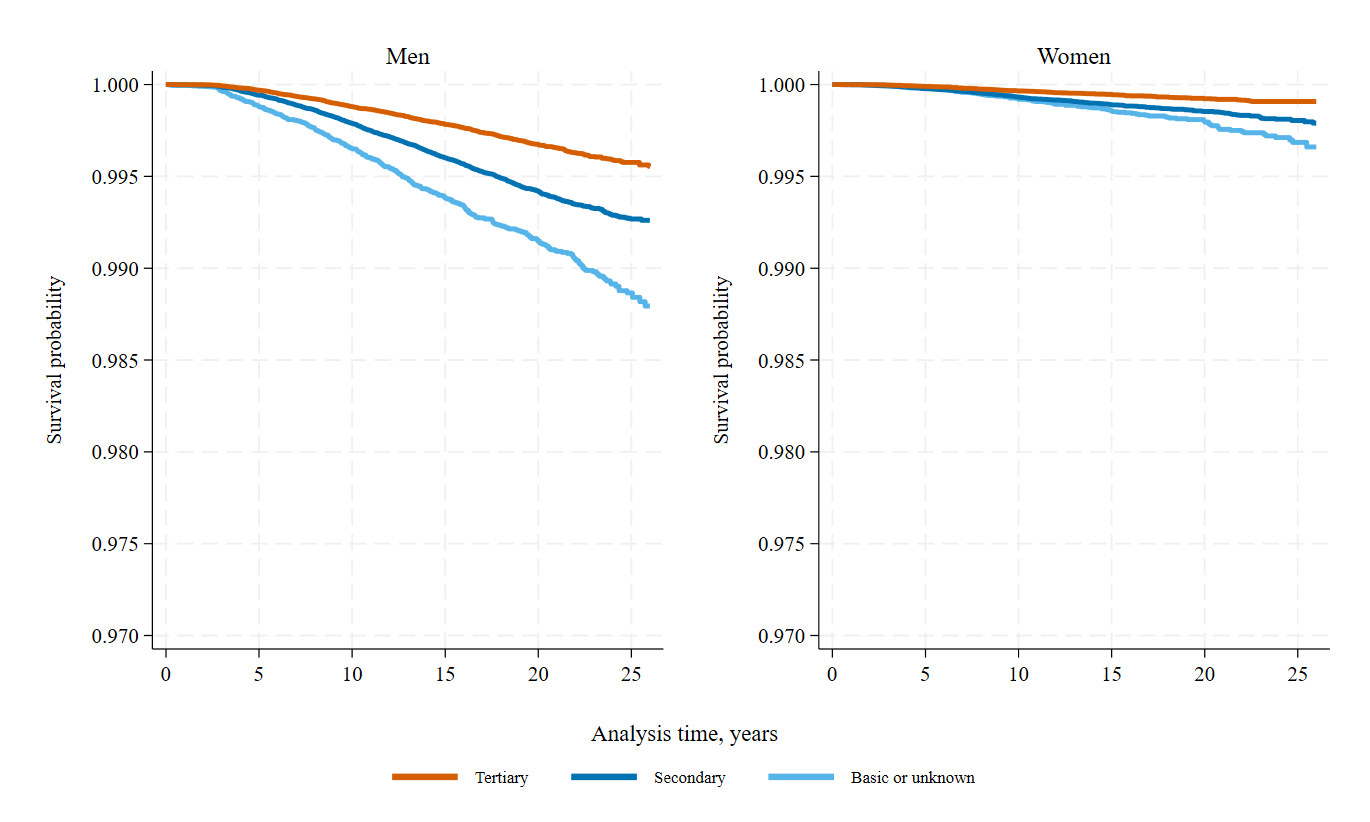


**Figure A1** Kaplan-Meier survival estimates by highest parental education for men and women.


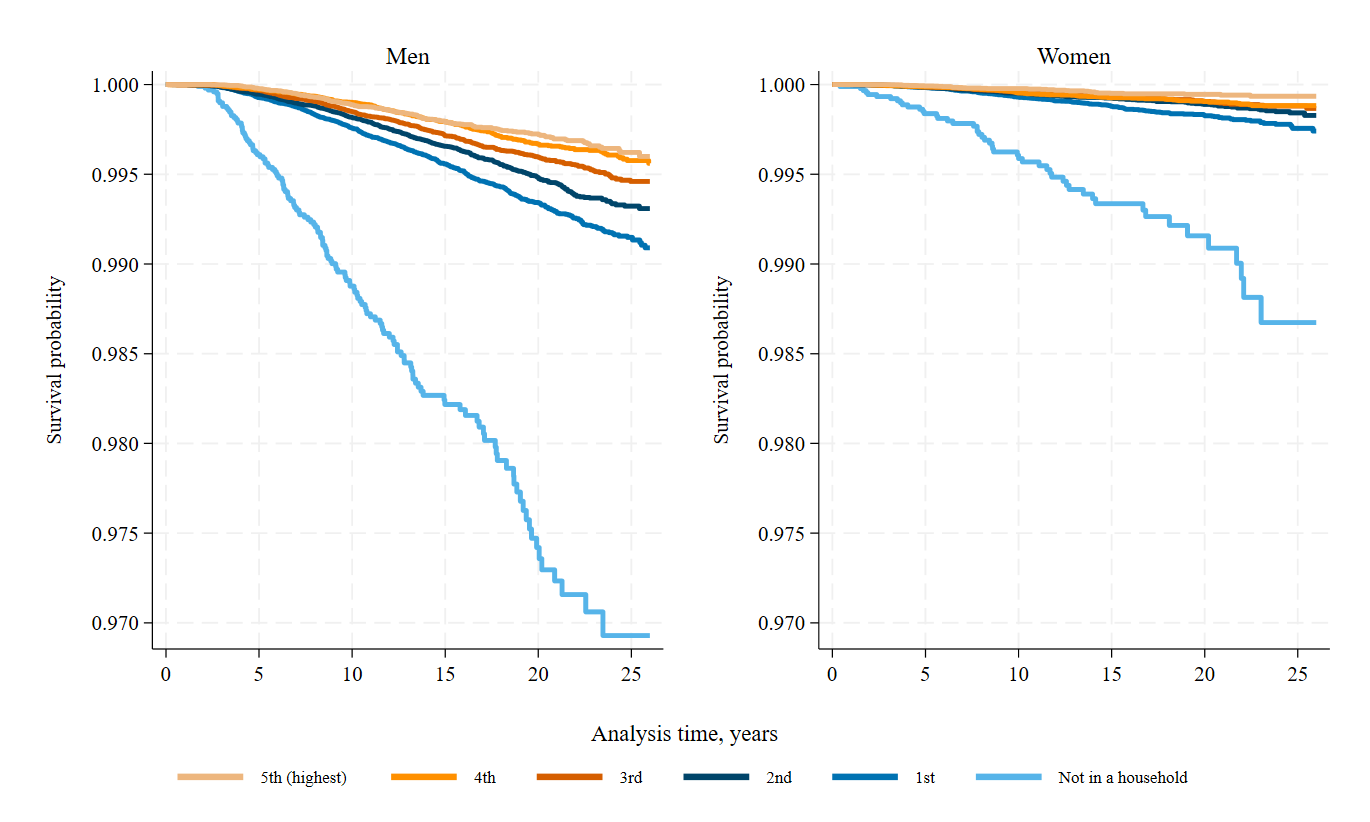


**Figure A2** Kaplan-Meier survival curves by household income quintiles for men and women.

**
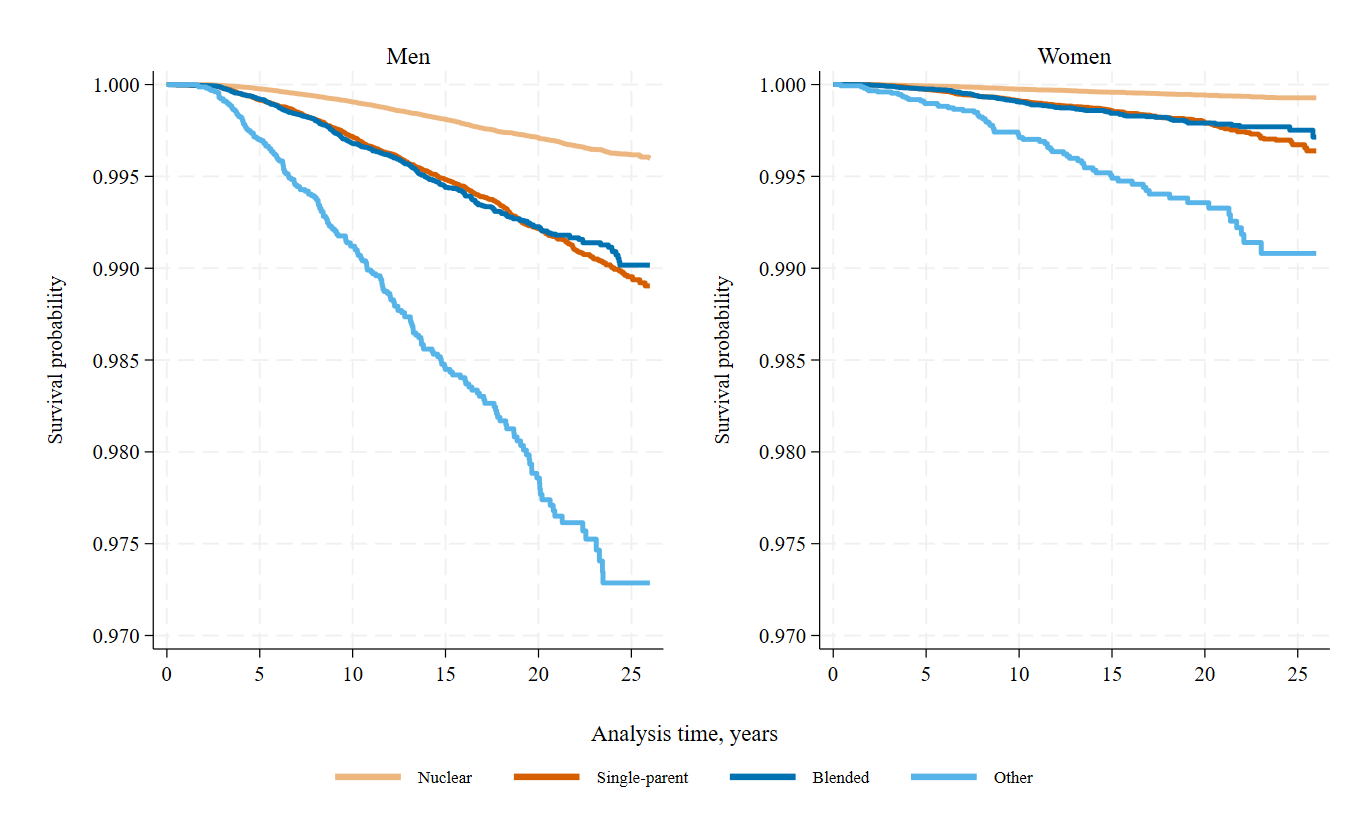
**

**Figure A3** Kaplan-Meier survival estimates by household type for men and women.

**
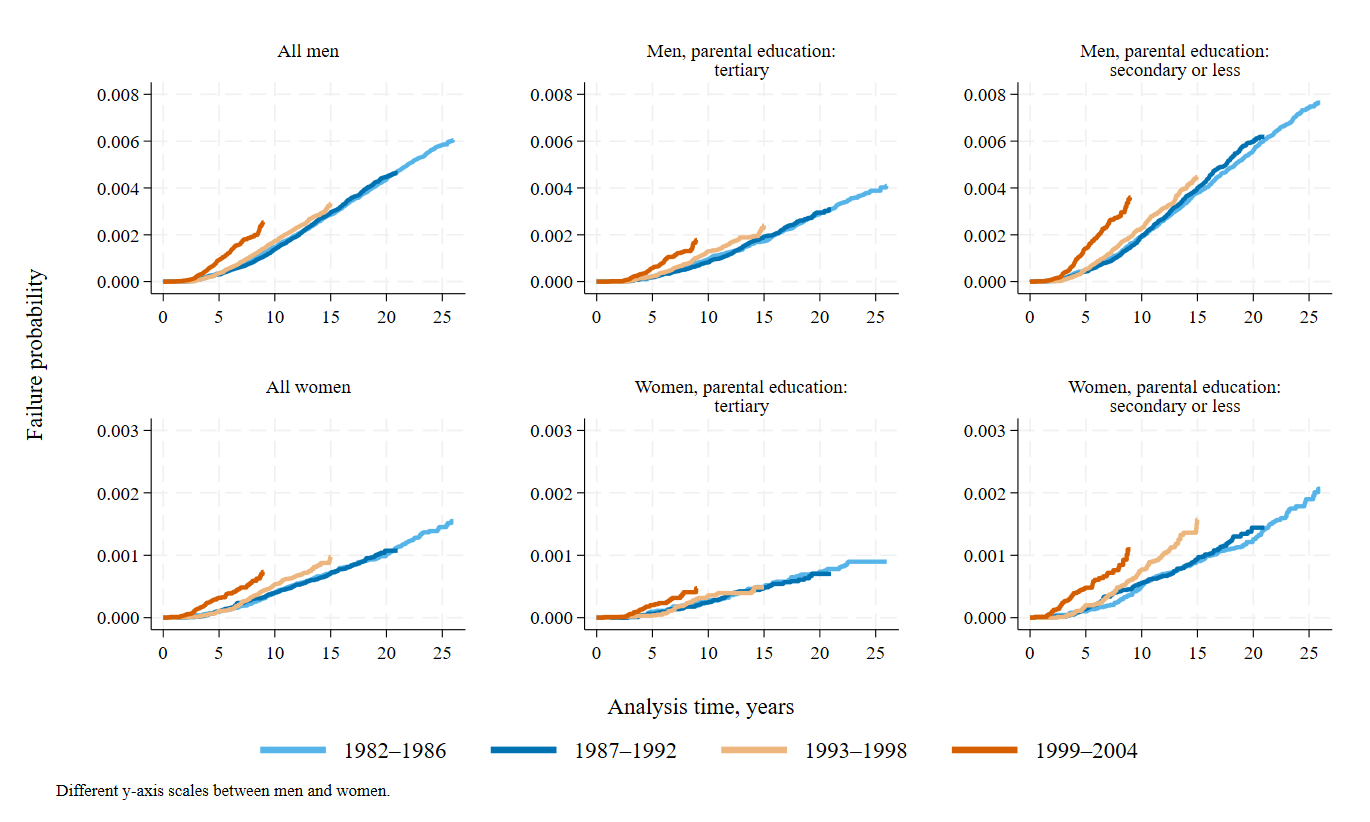
**

**Figure A4** Kaplan-Meier failure estimates by highest parental education and birth cohort for men and women. Population and drug-related deaths: cohort 1982–1986 (N=328 197, D=1 134); 1987–1992 (N=391 004, D=962); 1993–1998 (N=375 912, D=606); 1999–2004 (N=351 435, D=341).


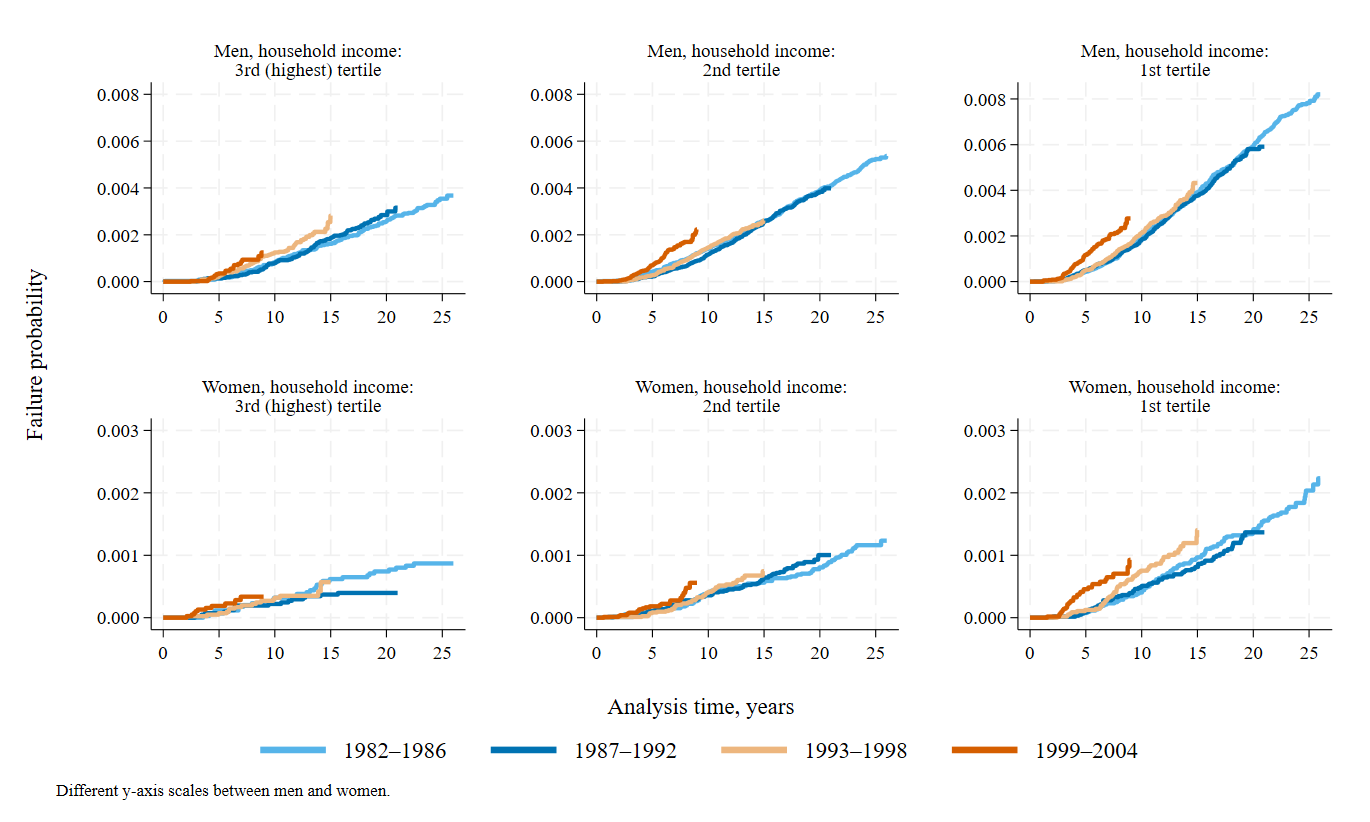


**Figure A5** Kaplan-Meier failure estimates by household income and birth cohort for men and women. Population and drug-related deaths: cohort 1982–1986 (N=328 197, D=1 134); 1987–1992 (N=391 004, D=962); 1993–1998 (N=375 912, D=606); 1999–2004 (N=351 435, D=341).


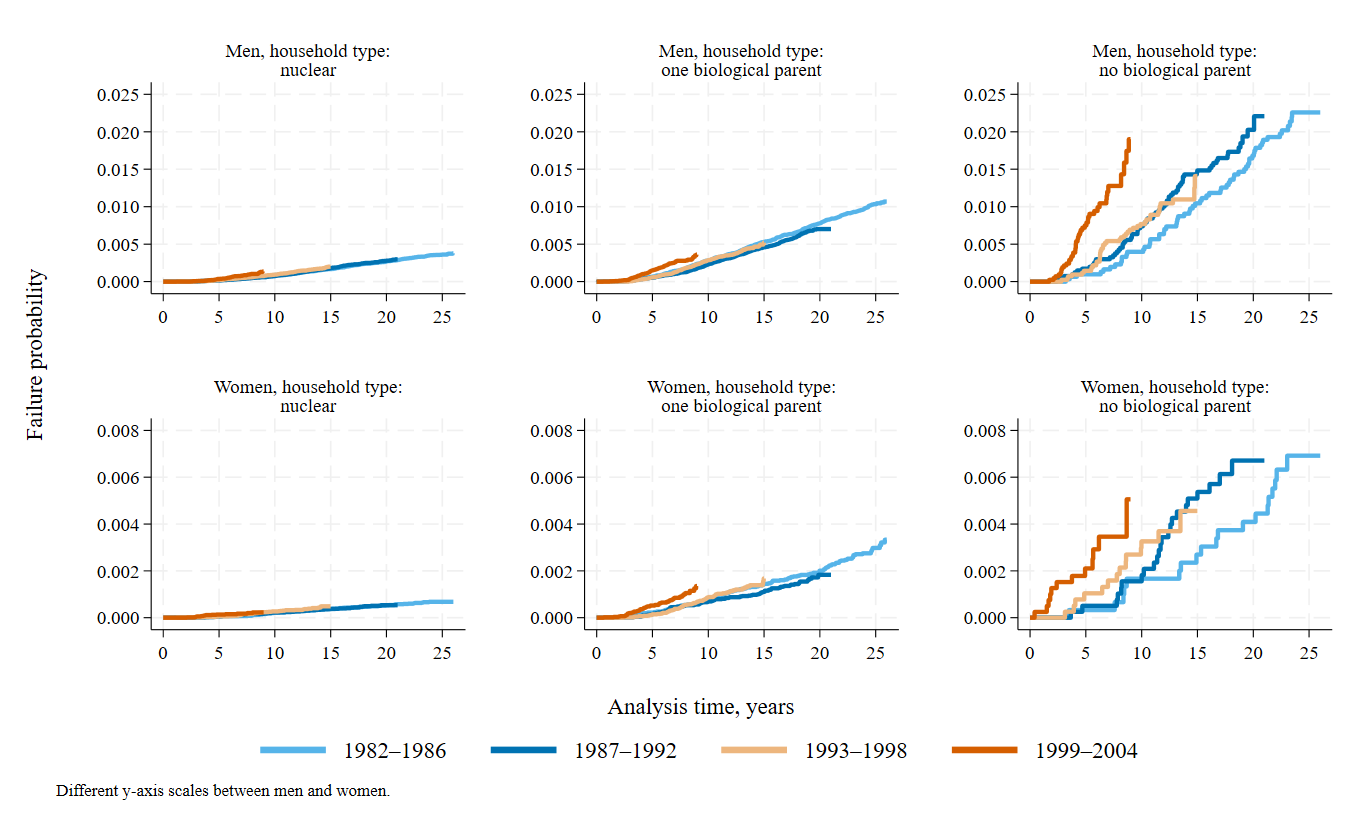


**Figure A6** Kaplan-Meier failure estimates by household type and birth cohort for men and women. Population and drug-related deaths: cohort 1982–1986 (N=328 197, D=1 134); 1987–1992 (N=391 004, D=962); 1993–1998 (N=375 912, D=606); 1999–2004 (N=351 435, D=341).

**
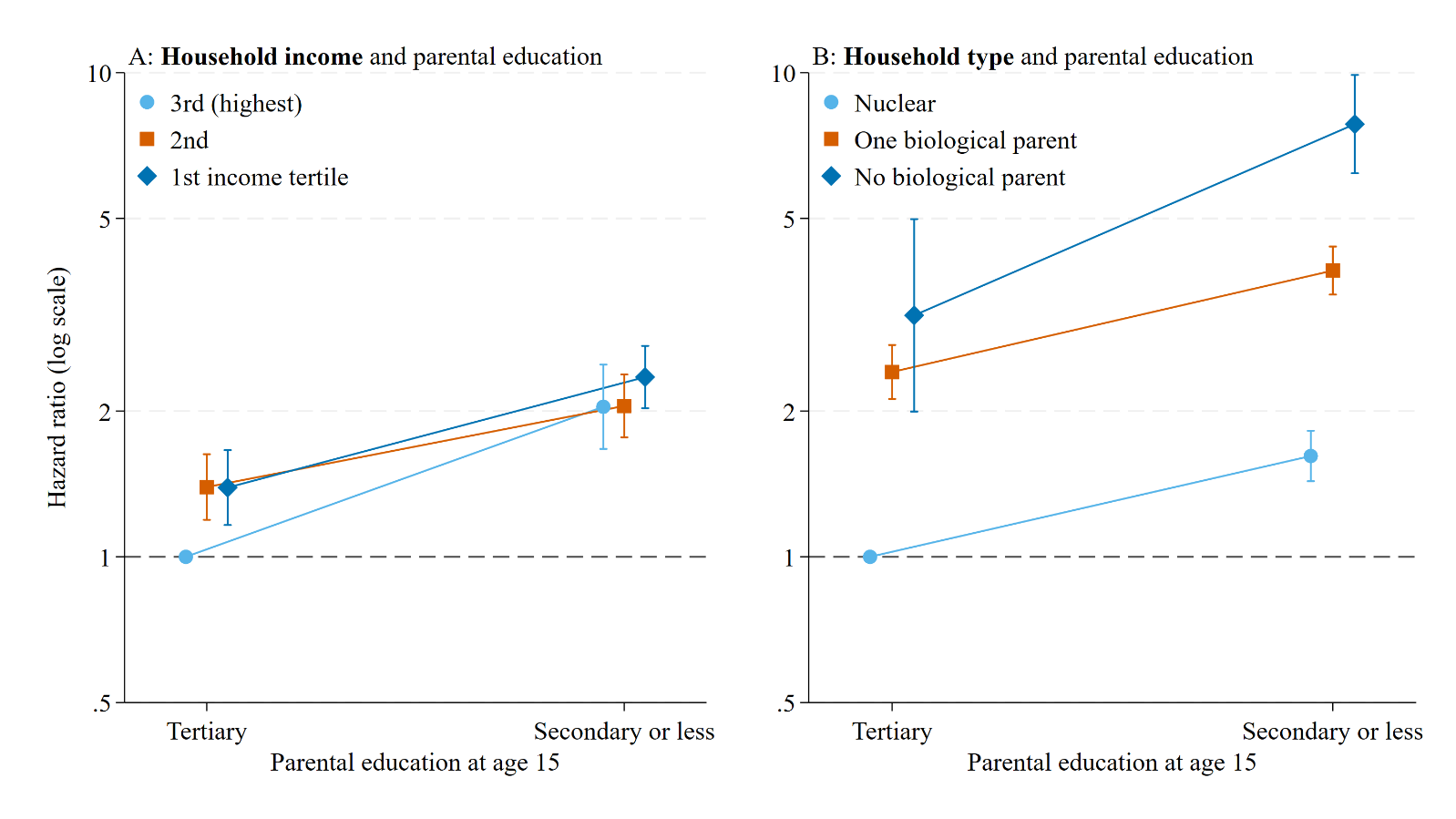
**

**Figure A7** Hazard ratios (log scale) of the interaction between household income and parental education (panel A) and household type and parental education (panel B) for drug-related mortality with 95% confidence intervals for combined men and women. The Cox regression models were adjusted for the household type (A)/household income (B), year of birth, sex and urbanicity. In the first interaction model (A) children outside of dwelling population were excluded (Household income: ‘Not in a household’). Reference category (A): at least one biological parent with tertiary education in a household belonging to the 3^rd^ (highest) income group. Reference category (B): at least one parent with tertiary education and child lives with both biological parents (nuclear family).

# References

1. EUDA. Statistical Bulletin 2025 — methods and definitions for drug-induced deaths [Internet]. 2025 [cited 2026 Feb 19]. Report No. Available from: https://www.euda.europa.eu/data/stats2025/methods/drd_en

2. Statistics Finland. Causes of death, national time series classification 2021 [Internet]. 2021 [cited 2025 Apr 2]. Available from: https://stat.fi/en/luokitukset/kuolinsyyt/kuolinsyyt_1_20210101
